# Pain management strategies for neuropathic pain in Fabry disease - a systematic review

**DOI:** 10.1186/s12883-016-0549-8

**Published:** 2016-02-24

**Authors:** Y. Schuller, G. E. Linthorst, C. E. M. Hollak, I. N. Van Schaik, M. Biegstraaten

**Affiliations:** Department of Internal Medicine, Division Endocrinology and Metabolism, Academic Medical Centre, Room F5-166, Meibergdreef 9, Amsterdam, 1105 AZ The Netherlands

**Keywords:** Fabry disease, Acroparesthesias, Neuropathic pain, Pain medication, Antiepileptics

## Abstract

**Background:**

Neuropathic pain is one of the key features of (classical) Fabry disease (FD). No randomized clinical trials comparing effectiveness of different pain management strategies have been performed. This review aims to give an overview of existing pain management strategies.

**Methods:**

PubMed and Embase were searched up to September 2014 for relevant articles on treatment of neuropathic pain in FD.

**Results:**

Seven-hundred-thirty-one articles were identified of which 26 were included in the analysis. Studies reported on 55 individuals in total, with group-sizes ranging from 1 to 8. Carbamazepine appeared most beneficial: complete pain relief in 5/25, partial relief in 17/25, and no benefit in 3/25 patients. Phenytoin resulted in complete relief in 1/27, partial relief in 12/27 and no benefit in 6/27 patients. In 8 patients a significant reduction in the frequency of pain attacks was described. Gabapentin caused partial relief in 6/7 and no relief in 1/7 patients. Little evidence was reported for SSNRI’s or treatment combinations. Adverse-effects were reported in all treatment strategies.

**Conclusions:**

Only for carbamazepine, phenytoin and gabapentin there is evidence of effectiveness in neuropathic pain due to FD, but comparison of effectiveness between these drugs is lacking. In routine clinical practice adverse-effects may discourage use of carbamazepine and phenytoin in favor of second-generation antiepileptic drugs, but this is currently not supported by clinical evidence. This review suffers greatly from incomplete outcome reports and a predominance of case reports, which emphasizes the need for robust clinical trials and observational cohort studies.

## Background

Fabry disease (FD, OMIM 301500) is a rare X-linked inherited lysosomal storage disease caused by a deficient or decreased activity of the lysosomal enzyme α-galactosidase A, as a result of a mutation in the GLA gene. The consecutive accumulation of glycosphingolipids, mainly globotriaosylceramide (Gb3), in lysosomes of several cell-types results in kidney, heart and nervous system complications [1]. FD is a heterogeneous disease with phenotypes ranging from severe, ‘classical’ FD to the more attenuated ‘non-classical’ form of the disease. Classically affected patients usually present at an early age with neuropathic pain, hypo- or anhidrosis, disseminated angiokeratoma, cornea verticillata, and microalbuminuria. At a later age, progressive damage to kidney, heart and brain may occur [2]. Patients with a ‘non-classical’ phenotype often have milder disease, and signs or symptoms may be limited to only one organ. The estimated prevalence of classical FD is 1 in 40.000 live births [3]. When non-classical and other GLA variants late-onset variants are considered, the prevalence may be as high as 1:1250 [4].

Neuropathic pain is one of the key features of the classical phenotype of the disease and has been shown to start on average at an age of 9 years in male patients and 16 years in female patients [5], but has even been reported in children as young as 2 years of age [6]. The pathophysiology of pain in FD is still poorly understood. Small fiber neuropathy (SFN) as a result of glycolipid accumulation in either the dorsal root ganglia or the endothelial cells of the blood vessels supplying the nerve fibers have been proposed as possible mechanisms [7]. Others have hypothesized that lysoGb3 (globotriaosylsphingosine, a deacylated Gb3 molecule) may exert a direct pathological effect on the ganglia or axons of Aδ fibers [8]. This hypothesis is supported in a recent study where a direct link between lysoGb3, increased intracellular Ca^2+^ levels in peripheral sensory neurons and pain was shown [9]. Whether this is causally related to small nerve fiber damage remains unclear [9].

Two types of pain often co-occur in classical FD: chronic pain in hands and feet and severe episodic pain attacks, also referred to as ‘Fabry crises’ [10, 11]. The latter are usually triggered by sudden changes in environmental or body temperature, and may persist for minutes to weeks [12]. The chronic pain is often described as burning, shooting or tingling pain, with a low to severe intensity. Both types of pain have been reported to be major causes of morbidity during the first 2 decades of life [13]. Moreover, research has shown that there is a strong association of chronic pain with depression [14]. As pain is a key feature in patients with FD an increased risk of depression is likely [15, 16]. It is therefore of importance to treat neuropathic pain adequately.

Treatment of neuropathic pain in FD starts with preventive measures and lifestyle changes, such as avoiding precipitating factors (physical exercise, exposure to sun or heat). Conservative therapy includes rest, holding icepacks, or administration of acetaminophen during febrile periods. Apart from these measures, additional pharmacological pain management is often necessary. A wide range of pain medication has been reported in the treatment of neuropathic pain in patients with FD. Gold et al. [15] report on a cohort of FD patients (*n* = 53) of whom 51 % uses pain medication, chronically. In a post-marketing registry study, 71 % of patients used analgesics, 36 % used anticonvulsants and 23 % used NSAIDs [17]. Furthermore the routine use of prophylactic medication has been reported in 23 patients including acetaminophen, carbamazepine, phenytoin and valproate [18]. Despite all the different analgesics that are prescribed in FD, the response to these regimens often remains unsatisfactory [19]. The current mainstay of therapy has been based on the long-term use of antiepileptics such as carbamazepine, gabapentin or phenytoin [20]. Nevertheless, little is known about its safety and effectiveness in patients with FD as no large-scale clinical trials or cohort studies have been performed to date. As a result, most data available come from small, uncontrolled, observational studies on less than 10 patients each. Here we report the results of a systematic review on the effectiveness and the adverse effects of different pain management strategies to treat neuropathic pain in FD. Our findings could serve as a rationale for controlled trials. We also aimed to develop a treatment algorithm for chronic and acute pain management in patients with FD.

## Methods

### Literature search

PubMed and Embase were searched from 1947 until September 2014 for relevant studies and reports on pain management strategies for neuropathic pain in FD. Key terms used were ‘Fabry disease’ and ‘pain’, including alternative notations. Also, ClinicalTrials.gov was searched to identify additional published or unpublished data. Additional reports were identified by hand searching the reference lists in the retrieved papers.

### Study selection

We included clinical trials, case series and case reports on the effect of pain medication in children as well as adults with FD and neuropathic pain. Studies on the effect of ERT on pain and reviews were not included in this review. Furthermore, studies lacking data on outcome were excluded. Articles reporting only on analgesics (acetaminophen and NSAIDs) were excluded, because these are known to be ineffective in the treatment of neuropathic pain in FD and the renal involvement due to FD makes NSAIDs unsuitable for chronic use in these patients. Title and abstract of all identified studies were read. If considered relevant, full text was read and analyzed. Data on pain severity and number of patients treated, drug and dosing regimen, study design, study duration and follow up, outcome measures and results, withdrawals and adverse events were extracted by one author (YS). As primary outcome we recorded chronic pain reduction after any treatment period as assessed in each study. Pain reduction was classified as complete relief of pain, partial relief of pain and no effect on the scale used in each study. As secondary outcome measures we recorded i) reduction of the frequency of pain attacks as reported by the patient, ii) any pain related outcome indicating improvement or worsening, iii) treatment withdrawals due to lack of effect, and iv) any (serious) adverse event while on treatment.

## Results

### Literature search results

**Fig. 1:**
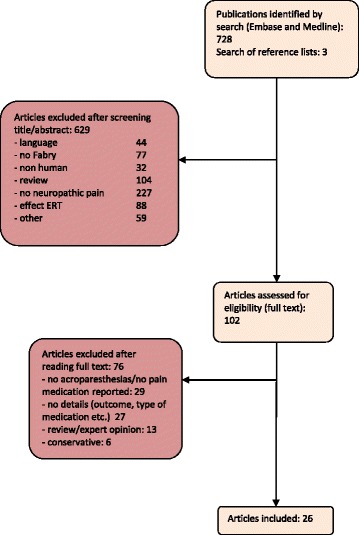
Study flow diagram. Numbers of studies screened, assessed for eligibility, and included in the review

The search provided 728 articles (Fig. 1, study flow diagram). Three additional articles were found by searching the reference lists [17, 21, 22]. We excluded 629 articles after screening of title and abstract. One hundred and two articles were read in full text, of which 76 were subsequently excluded. Forty-eight of these 76 articles were considered to be less relevant, because referrals to other studies were made, no information was provided about analgesic treatment, or only conservative pain management (e.g., rest, holding ice packs) was described [11, 18, 20, 23–38]. Twenty-seven articles provided information on pain management, but details on outcome were lacking. One case report describing a male patient using carbamazepine was excluded [39]. This 34-year old male carried a GLA variant (A143T) which is generally considered a polymorphism based on normal biopsy results and normal lysoGb3 levels in individuals carrying this variant [40–42]. He had a 5-year history of activity-induced foot and leg cramps and fasciculations with pain, which is not a typical presentation of FD neuropathic pain, while the intraepidermal nerve fiber density, kidney biopsy and cardiac and brain investigations were all normal. Taking the controversial mutation and his clinical picture together, we considered his symptoms not to be caused by FD.

Twenty-six studies were included in the final analysis and these reported on 55 patients in total [21, 43–67]. Patients were 3-45 years old, and at least 15 patients were below 18 years of age. An exact number could not be calculated due to the lack of data in the majority of included studies. Case-reports predominated; only 7/26 reports included more than 1 patient. None of the studies were randomized controlled trials. Three studies were performed with the specific aim to evaluate the effectiveness of a treatment for neuropathic pain in FD [43, 49, 53]. Treatment group sizes were small, ranging from 1 to 8 patients per report. A variety of different pain assessment tools was reported, including visual analogue scales (VAS) (4 %), the brief pain inventory (BPI) (4 %), pain relief scores (8 %), or other subjective pain scoring tools (84 %). The majority of studies (84 %) did not specify study duration. Where mentioned, most studies were of relatively short duration: individual treatment periods ranged from several days (case reports) to up to 5 years (observational studies). Details of included studies are reported in Table 1.

**Table 1:** Results of individual studies. This table gives an overview of the characteristics of each study included in the review, and summarizes the main endpoints

Study (N, sex)	Medication	Dose	Effectiveness recorded chronic pain reduction after any treatment period		Adverse events
			As reported in study	As rated for this review	
Monotherapy – primary endpoint					
Patil [52] (*N* = 1, male)	Carbamazepine	Not reported	Some response	Partial pain relief	Not reported
Politei [67] (*N* = 2, 1 male)	Carbamazepine	600 mg/day	Good response	Complete pain relief (*N* = 1)	Not reported
		?	No response	No response (*N* = 1)	
Lim [48] (*N* = 1, male)	Carbamazepine (+ERT)	600 mg/day	Condition stationary	Partial pain relief	Not reported
Tümer [58] (*N* = 1, female)	Carbamazepine	10 mg/kg/day (23 kg)	Pain resolved completely	Complete pain relief	Not reported
Chaudhuri [60] (*N* = 1, male)	Carbamazepine	Not reported	Pain reasonably controlled	Partial pain relief	Not reported
Mills [65] (*N* = 3, males)	Carbamazepine (+ERT)	Patient 1: 200 mg eod	60 % improvement on questionnaire on double dose of ERT	Partial pain relief	Not reported
		Patient 2: 200 mg/day	Subjective improvement (25 % improvement on questionnaire on double dose of Fabrazyme)	Partial pain relief	
		Patient 3: 200 mg/day	Absence of breakthrough pain, questionnaire results improved by over 90 %	Partial pain relief	
Yang [59] (*N* = 1, male)	Carbamazepine	200-400 mg/day	Pain controlled	Partial pain relief	Not reported
Asahi [61] (*N* = 1, male)	Carbamazepine	Not reported	Complete pain relief	Complete pain relief	Not reported
Slee [55] (*N* = 1, male)	Carbamazepine	600 mg/day	Pain controlled	Partial pain relief	Not reported
Brady [62] (*N* = 1, male)	Carbamazepine	Not reported	Considerable relief from painful acroparesthesias	Partial pain relief	Discontinued due to drowsiness
Shelley [63] (*N* = 1, male)	Carbamazepine	Not reported	Modest relief	Partial pain relief	Not reported
Inagaki [46] (*N* = 1, male)	Carbamazepine	300-500 mg/day	Partially helpful in alleviating shooting pain	Partial pain relief	Not reported
Filling-Katz [43] (*N* = 7, males)	Carbamazepine	0.8-15.9 mg/kg/day	Partial amelioration in 3 patients (43 %), complete pain relief in 2 patients (29 %), no benefit in 2 patients (29 %)	Complete pain relief (*N* = 2)	Autonomic complications in 2/7 patients (27 %), discontinuation in 1 patient.
				Partial pain relief (*N* = 3)	
				No effect (*N* = 2)	
Tomé [57]+ Lenoir [47, 57] (same patient) (*N* = 1, male)	Carbamazepine	600 mg/day	Pain attacks almost disappeared	Partial pain relief	Not reported
Shibasaki [54] (*N* = 1, male)	Carbamazepine	200 mg/day	Pain suppressed	Partial pain relief	No side effects
Gordon [44] (*N* = 1, male)	Phenytoin	5 mg/kg/day	Ineffective	No effect	Discontinuation
Paira [50] (*N* = 1, male)	Phenytoin	300 mg/day	Pain controlled	Partial pain relief	Not reported
Filling-Katz [43] (*N* = 5, males)	Phenytoin	Therapeutic dosage (mean 13 mg/dl)	Inadequate pain control (5/5 patients)	No effect (*N* = 5)	Not reported
Sheth [64] (*N* = 2, 1 male)	Phenytoin	Not reported	Pain controlled in both patients	Partial pain relief (*N* = 2)	Not reported
Duperrat [66] (*N* = 1, male)	Phenytoin	200 mg/day	Pain completely disappeared	Complete pain relief	Not reported
Lockman [49] (*N* = 8, 7 males)	Phenytoin	300 mg/day or 4-6 mg/kg/day	Average pain relief score of 2,7 (complete pain relief, *p* < 0,001 when compared to ASA or placebo)	Partial pain relief (*N* = 8)	Dizziness, drowsiness and headache in 1 patient
Shibasaki [54] (*N* = 1, male)	Phenytoin	200 mg/day	Ameliorated pain	Partial pain relief	Not reported
Park [51] (*N* = 1, male)	Gabapentin	Not reported	Pain persisted	No effect	Not reported
Ries [11, 53] (*N* = 6, males)	Gabapentin	Average daily dose 917 mg (range 100-1200 mg)	Average pain scores decreased from 5.0 (range 4-6) to 3.7 (range 3-6) with an intraindividual reduction of 1.3 (range 0-3) (*p* = 0,22)	Partial pain relief (*N* = 6)	Generally well tolerated. Vertigo and blurred speech in 1 patient
Inagaki [46] + Inagaki [45] (same patients) (*N* = 2, males)	Neurotropin	4 units (crisis)	Pain almost completely eliminated	Partial pain relief (*N* = 2)	Not reported
Wise [21] (*N* = 2, males)	Pethidine	500-700 mg/day i.m.	Pain reasonably controlled	Partial pain relief (*N* = 2)	Not reported
Politei [67] (*N* = 2)	Lidocaine	2 mg/kg i.v.	Quick pain relief in pain crisis	Partial pain relief (*N* = 2)	
Combination therapies – primary endpoint					
Park [51] (*N* = 1, male)	Phenytoin + Carbamazepine	200 mg/day + 400 mg/day	Mild-moderate pain persisted	Partial pain relief	Not reported
	Phenytoin + Carbamazepine (+ERT)	100 mg/day + 200 mg/day	Pain decreased	Partial pain relief	
Gordon [44] (*N* = 1, male)	Morphine + Amitriptyline (crisis)	0.06 mg/kg IV push, 0.02 mg/kg/hr IV; 0.25 mg/kg p.o. at bedtime	Pain control within hour, remaining pain free overnight	Complete pain relief	Not reported
Inagaki [46] + Inagaki [45] (same patients) (*N* = 2, males)	Neurotropin + Carbamazepine	12-16 units/day + 600 mg/day	Pain disappeared almost completely	Partial pain relief (*N* = 2)	Not reported
Monotherapy - Secondary endpoints					
Gordon [44] (*N* = 1, male)	Carbamazepine	?	Reduced frequency and duration of crises (to 3-4 times annually)	Partial pain relief (*N* = 1)	Not reported
Spence [56] (*N* = 8, males)	Phenytoin	100-400 mg/day	Significant reduction in frequency of painful crises in 7/8 patients	Partial pain relief (*N* = 8)	Not reported
				Unknown (*N* = 1)	

Abbreviations: *ERT* enzyme replacement therapy, *eod* every other day, *CMZ* carbamazepine, *p.o.* per os

Note: some studies are mentioned more than once due to use of several pain management strategies

Results stating ‘pain controlled’ interpreted by authors as ‘partial pain relief’

### Pain management strategies and effects

The following analgesics were used: carbamazepine, gabapentin, phenytoin, neurotropin and opioids Results of all pain strategies are summarized in Table 1.

Carbamazepine was used in 27/55 patients (49 %, reported in 18 studies), most often as mono-therapy (25 patients, 44 %, reported in 17 studies). Patients used carbamazepine dosages of 100-600 mg/day, or 0.8-15.9 mg/kg/day. Complete relief of pain was described in 5 of the 25 patients on mono-therapy [43, 58, 61, 67], partial relief in 16 patients [43, 46–48, 52, 54, 55, 57, 59, 60, 62, 63, 65] and no effect in 3 [43, 67]. Additionally, 1 patient reported a reduced frequency and duration of crises. Four of the 25 patients were treated simultaneously with enzyme replacement therapy (ERT) and all showed partial pain relief. The effect of carbamazepine was confirmed by a double-blind cross-over design in one patient [54]. It was started with a daily dosage of 200 mg, which was enough to suppress the pain. Substitution by placebo was followed by a reappearance of the excruciating pain within 48 h, and readministration of the drug relieved the pain completely. In the same patient, the effect appeared faster and lasted longer than in the case of phenytoin [54]. Oral administration of carbamazepine was only partially helpful in alleviating the shooting pain in one patient (reported in two studies), but after treatment with neurotropin orally in addition to carbamazepine, the constant paresthesia and episodic shooting pain disappeared almost completely [45, 46]. Other combination strategies resulted in complete pain relief with gabapentin and carbamazepine in 1 patient [45]. Gabapentin alone was ineffective in a study by Park et al., but it should be noted that it was discontinued after just 2 days. Subsequently, a combination of phenytoin and carbamazepine was administered, which caused partial pain relief [51]. Gabapentin monotherapy has been described in one other study in which 6 patients were included. All of these patients showed a partial relief of pain on an average daily dose of 917 mg [53].

In a study by Filling-Katz, 5 of 7 patients on carbamazepine therapy had a history of phenytoin use [43]. Phenytoin at therapeutic dosages provided inadequate pain control by patient report in all 5 subjects. The effect of phenytoin was reported in an additional 22 patients (in 7 studies), with a dose range of 100-400 mg/day. Complete pain relief was achieved in 1 patient, and partial pain relief in 12 patients. Phenytoin was ineffective in 6 patients, even though treatment duration ranged from 6 months to 5 years [43]. In 8 patients a significant reduction in the frequency of pain attacks was described [56].

One study published in 1962 described the use of pethidine in 2 male patients, in whom partial pain relief was achieved with 500-700 mg/day IM [21]. Another study reports beneficial effect on pain during a crisis with intravenous lidocaine [67].

### Adverse effects

Three out of 20 studies including 4 patients reported on adverse effects of carbamazepine. Dose-related autonomic complications were reported in 2 patients, and necessitated dose reduction in 1 patient and discontinuation in the other [43]. Carbamazepine was discontinued because of drowsiness in 1 patient [62]. One patient had no adverse effects of carbamazepine [54]. Vertigo and blurred speech were reported in 1 patient treated with gabapentin, which disappeared upon dose reduction [53]. In the same study, it was stated that gabapentin was tolerated generally well in the other patients. The other study on patients using gabapentin did not report on adverse effects. Only 1 out of 8 studies in which phenytoin was used (*n* = 27 patients) reported on adverse effects. In this study 1 patient suffered from dizziness, drowsiness and headache [49]. Furthermore, discontinuation was reported for phenytoin because of poor compliance in 1 patient [44]. Few adverse effects (not further specified) were reported on the combination of neurotropin with carbamazepine in 1 patient [45].

## Discussion

This systematic review of the literature on neuropathic pain management in FD clearly demonstrates that currently available effectiveness data are mainly derived from case reports and small observational studies. This is disappointing when considering the high prevalence of chronic pain in patients with FD and the fact that analgesics are frequently prescribed by caregivers. Apparently, most physicians treating patients with FD have local procedures, based on either treatment protocols for painful neuropathies not specific for FD, or personal experiences. Of these drugs, anti-epileptics, including carbamazepine, gabapentin and phenytoin appear to be prescribed most often.

Based on this review we conclude that there is class IV evidence that carbamazepine and phenytoin are effective in the treatment of FD neuropathic pain, although it remains unclear to which extent. This is in agreement with previous reviews reporting on drug use for neuropathic pain in FD [37, 68]. For amitriptyline, pregabalin and lamotrigine there appears to be no evidence, insufficient evidence, or even evidence of a lack of effect. For gabapentin there was so little evidence that no sensible judgment could be made about its effect on neuropathic pain in FD patients, even though it is frequently prescribed in patients with FD. Adverse-effects were seen in all reported drugs, varying from dizziness (phenytoin), to vertigo (gabapentin) and autonomic complications (carbamazepine).

### Implications for practice

As is evident from this review, the literature does not provide an answer to the important pragmatic question which drug should be prescribed for the treatment of neuropathic pain in FD. In addition, there is no sound evidence in which order drugs should be evaluated. Clinical practice has shown that most patients may achieve good results with carbamazepine or phenytoin. Phenytoin, however, is known to be associated with a number of potentially troublesome adverse events, including neurologic and hematologic effects [69]. In addition, there is an increased risk of teratogenicity for both carbamazepine and phenytoin. The teratogenic potential of the newer antiepileptic drugs and antidepressants in still unclear; small sample sizes and exposure to multiple drugs have precluded a definite conclusion so far. It is therefore important to discuss carefully and tailored to each patient, the benefit and risks of each drug for mother and fetus [70–72]. Both carbamazepine and phenytoin are known to be broad-spectrum enzyme inducers by stimulating the activity of many cytochrome P450 (CYP) enzymes (CYP2B6, CYP2C9, CYP3A4 and CYP1A2) [73]. They can therefore reduce the effectiveness of several co-administered medications, such as statins (simvastatine, atorvastatine), SSNRI’s (duloxetine, venlafaxine), (dihydropyridine) calcium antagonists (e.g., amlodipine, nifedipine), angiotensin receptor blockers (ARBs) (losartan, candesartan, irbesartan), anticoagulants (warfarin) and steroids [74]. Taking into account that the incidence of kidney- and heart-related comorbidities is high in FD patients, interaction with calcium antagonists and ARBs is inconvenient.

Vice versa, other commonly prescribed drugs in patients with FD (fluoxetine, verapamil, amiodarone) may increase the serum concentration of phenytoin and carbamazepine by inducing CYP enzymes [75]. In women with neuropathic pain, it is important to note that carbamazepine, as well as phenytoin, increase the clearance of contraceptive hormones, which may lead to contraceptive failure. The use of carbamazepine and phenytoin is further limited by their potential to cause undesirable side-effects, such as gastrointestinal complaints and cytopenia. In addition, the need for laboratory monitoring (sodium, leukocytes, transaminases) in patients on carbamazepine resulted in preference for newer antiepileptic drugs, such as gabapentin and pregabalin. These are excreted unchanged through the kidneys with no reliance on liver metabolism. Therefore they do not lead to stimulation of the CYP enzymes and lower concentrations of other drugs as carbamazepine does [76]. On the other hand, the excretion by the kidneys results in the need for a dose reduction in renal insufficiency, one of the features of FD [77]. Since pharmacokinetics of gabapentin are nonlinear, dosing requires careful titration [76]. Pregabalin has pharmacokinetic advantages to gabapentin as it has linear pharmacokinetics. Consequently, dosing is more straightforward and requires only a twice daily administration [78]. The effectiveness and tolerability of pregabalin seem to be similar to those of gabapentin. Both drugs are fairly well tolerated. Dose-dependent dizziness and sedation can easily be reduced by starting with lower dosages and dose titration. However, although pregabalin and gabapentin are widely prescribed and effective for neuropathic pain in general, little is known about their effect in FD neuropathy [79].

Little has been reported about the use of dual serotonin and norepinephrine reuptake inhibitors such as venlafaxine and duloxetine in treating neuropathic pain due to FD. Duloxetine has shown consistent effectiveness in painful diabetic peripheral neuropathy, with effectiveness sustained for 1 year in an open-label trial [80]. Unfortunately, duloxetine has not been studied in other types of neuropathic pain, and so its effectiveness in such conditions is unknown. Besides, both duloxetine and venlafaxine have potential concomitant and undesirable adverse-effects in patients with FD because of their anti-cholinergic effect (e.g., constipation, anhydrosis, palpitations) [68]. Finally, opioids are shown to be effective in the treatment of painful crises [44], but it should be noted that the chronic use of opioids may cause obstipation, dependence, drowsiness and involves the risk of substance abuse.

Most of the medications we have discussed provide only partial pain relief, and adverse effects may limit dose escalation. Hence, in clinical practice, it would make sense to use 2 or more medications in combination in order to achieve either an additive beneficial effect or a reduction in the adverse effects associated with the use of a high dose of a single drug. However, little evidence is available to support the use of such combinations. Recently, combination therapy is advised if at least two monotherapies failed to relieve pain in FD [37]. A treatment algorithm is proposed, which advises second generation antiepileptics (pregabalin, gabapentin) to be used as medicines of first choice. Drugs of second and third choice were phenytoin, carbamazepine, and duloxetine. On the contrary, a report of an expert panel on the treatment of neuropathic pain in FD recommends carbamazepine as the drug of first choice. Anticonvulsants and antidepressants are also considered viable options [68]. Due to the limited amount of evidence with respect to the effectiveness of pain medication in FD, clinicians are often forced to rely on general neuropathic pain protocols. In a recently published meta-analysis about pharmacotherapy for neuropathic pain in adults, which included 229 studies, a strong recommendation was given for tricyclic antidepressants (amitriptyline), serotonin-noradrenaline reuptake inhibitors (duloxetine, venlafaxine), pregabalin, and gabapentin as first line treatment for neuropathic pain. Recommendations about carbamazepine were inconclusive [81]. Another guideline for the pharmacological management of neuropathic pain recommends duloxetine, venlafaxine, gabapentin, pregabalin and topical lidocaine as drugs of first choice. Opioids are recommended as drugs of second choice, and carbamazepine as drug of third choice [76].

Since conditions that cause neuropathic pain in children are relatively uncommon, data on general neuropathic pain treatment in children are limited by small numbers and few randomized controlled trials, leaving clinicians with many unanswered questions regarding clinical practice [82]. It is unclear if generalization of interventions used for neuropathic pain in adults may be appropriate. Therefore, trials are needed on both the safety and efficacy of drugs for the treatment of neuropathic pain in children [82, 83]. Despite the paucity of evidence, a treatment algorithm for the treatment of neuropathic pain in children with FD was published. It discusses the use of carbamazepine, antidepressants and anticonvulsants and includes dosing and titration schemes [84].

Although evidence exists suggesting that some diseases causing neuropathic pain respond differently to the same medications [85], we can conclude that it seems inevitable to base a treatment algorithm for FD patients for the greater part on experiences with neuropathic pain in general.

Taking all these findings and considerations into account we developed a treatment algorithm for pain management in patients (adults as well as children) with FD at our hospital (Academic Medical Center, Amsterdam, The Netherlands). In line with the expert panel report [68], the included studies in this review and our own clinical experiences, carbamazepine is considered the drug of first choice. In case of treatment failure or contraindications, we consider second generation anti-epileptics to be proper drugs of second choice. Drugs of third choice include SSNRI’s such as duloxetine and venlafaxine. In Appendix 1, an overview of all agents is presented.

### Role of enzyme replacement therapy

Studies on the effect of ERT on neuropathic pain have shown conflicting results. Agalsidase alfa reduced the severity of neuropathic pain in a randomized controlled trial (RCT), but an imbalance in baseline pain scores hampers the interpretation of the results [86]. A RCT on the effect of agalsidase beta showed a significant reduction in pain in both the treatment and the placebo group [87]. Several observational studies showed no reduction of pain severity with ERT [87–90], while there is some evidence that ERT has a positive effect on pain in children [31, 91]. Altogether, it remains unclear whether ERT has an effect on the neuropathic pain in patients with FD. Recently published European recommendations for initiation and cessation of ERT in FD indicate that ERT should be considered in patients with neuropathic pain, and may even be considered if the pain is completely controlled with pain medication [2]. The formulation of this recommendation implies that the decision to start ERT should be made on an individual basis taking into account FD features and personal preferences. In clinical practice, physicians will often start with pain management, for which the current algorithm can be of help to try to achieve maximum response. However, for future research, it would be interesting to conduct a properly designed study to clearly evaluate the effectiveness of ERT with or without concurrent use of pain medication on neuropathic pain.

### Limitations

The predominance of case-reports in this review has to be considered as a source of bias and therefore caution is needed in interpreting the data. Meaningful comparison of effectiveness with other interventions is not possible. In addition, this review suffers greatly from the lack of details provided in the case reports and case series.

## Conclusion

Perhaps the most important conclusion of this review is the need to perform large, high quality, long duration studies using robust endpoints that actually measure effectiveness of different analgesic drugs in FD neuropathic pain. It would be of particular interest to compare first generation (carbamazepine, phenytoin) with second generation antiepileptics (pregabalin, gabapentin). Such a study would require a uniform group (preferably classically affected males), and an easily interpretable clinical endpoint (e.g., changes in VAS or Brief Pain Inventory score) for an extended period of time. As soon as such study results are published we will be able to update the currently presented treatment algorithm.

## Compliance with ethics guidelines

All authors declare that the article has not been submitted for publication elsewhere. All authors thoroughly inspected the manuscript and contributed equally. Every author agreed to submit the article in BMC Neurology.

## Appendix 1: Treatment options

A summary of the most commonly prescribed drugs in neuropathic pain in FD, with information on dosage, titration and precautions.

## Abbreviations

ARB: angiotensin receptor blocker
CYP: cytochrome p
ERT: enzyme replacement therapy
FD: Fabry’s disease
Gb3: globotriaosylceramide
GLA: galactosidase alpha
LysoGb3: globotriaosylsphingosine
NSAID: non-steroidal anti-inflammatory drug
SSNRI: selective serotonin-norepinephrine reuptake inhibitors
