# Erratum to: Pain management strategies for neuropathic pain in Fabry disease - a systematic review

**DOI:** 10.1186/s12883-016-0590-7

**Published:** 2016-05-16

**Authors:** Y. Schuller, G.E. Linthorst, C.E.M. Hollak, I.N. Van Schaik, M. Biegstraaten

**Affiliations:** Department of Internal Medicine, Division Endocrinology and Metabolism, Academic Medical Centre, Room F5-166, Meibergdreef 9, Amsterdam, 1105 AZ The Netherlands

## Erratum

After publication of the original article [1], it came to the authors’ attention that Additional file 1 had been erroneously included as Appendix 1. As a result, the published article did not contain an option to download the Additional file; ‘Treatment options: A summary of the most commonly prescribed drugs in neuropathic pain in FD, with information on dosage, titration and precautions’ is available to download as Additional file 1 in this erratum.

## Additional file

Additional file 1: Treatment options: A summary of the most commonly prescribed drugs in neuropathic pain in FD, with information on dosage, titration and precautions. (DOC 50 kb)
