# Supplementary material for: Erratum to: Pain management strategies for neuropathic pain in Fabry disease - a systematic review
Source: BMC Neurol. 2016 May 16;16:67. doi: 10.1186/s12883-016-0590-7 (PMC4869195; doi:10.1186/s12883-016-0590-7)
Supplement: Additional file 1: — Treatment options: A summary of the most commonly prescribed drugs in neuropathic pain in FD, with information on dosage, titration and precautions. (DOC 50 kb) [file 12883_2016_590_MOESM1_ESM.doc]

**Additional file 1: Treatment options**

| **Medication** | **Starting dosage** | **Titration** | **Maximum dosage** | **Duration of adequate trial** | **Warnings/precautions** |
| --- | --- | --- | --- | --- | --- |
| Carbamazepine | 200-400 mg/day in 2 divided doses | gradually increase in increments of 200 mg every day  (administration in 2-3 divided doses daily) | 1200 mg daily | 1-2 weeks | - Use with caution in patients with atrioventricular heart block or blood disorders  - Increases clearance of contraceptives. Women who use contraceptives are advised to use preparations containing at least 50 μg of ethinylestradiol, or switch to alternative contraceptive methods (Implanon, Depo-Provera, Mirena) in order to reduce the chance of pregnancy - High risk of teratogenicity. Cease therapy when pregnant. - Causes enzyme induction (CYP2C9, CYP3A and CYP1A2), which can interfere with other drug therapies (e.g. anticoagulants, calcium antagonists, angiotensin receptor blockers, statins, antihypertensives) - Monitor CBC, platelets, and differential prior to and during therapy; discontinue if significant bone marrow suppression occurs. - Use with caution in patients with hepatic impairment or history of hepatic porphyria.  - Has mild anticholinergic activity; use with caution in patients with sensitivity to anticholinergic effects - Han-Chinese or Thai patients with the variant HLA-B*1502 may be at an increased risk of developing Stevens-Johnson syndrome and/or toxic epidermal necrolysis. - Little or average negative influence on driving performance. Comparable to blood alcohol content of 0,5–0,8‰. Advice: doses below 600 mg/day: no driving in first week. Doses higher than 600 mg/day: no driving until 1 year after initiation of therapy. |
| Phenytoin (unlabeled use) | 150-200 mg/day in 2 divided doses | *Small changes in doses can cause large changes in serum levels!* Check phenytoin serum levels after 7-10 days and adjust dose if necessary  Rough guide to making an adjustment to the daily dose:  - concentration <7 ug/mL 🡪 increase dose by 100 mg/day  - concentration 7-12 ug/mL 🡪 increase dose by 50 mg/day - concentration >12 mg/mL, increase dose by 30 mg/day - concentration >16 ug/mL 🡪 any change may result in significant increase in serum level and should be done very carefully. - toxic phenytoin level:  > 20 ug/ml | 400 mg/day | 2-5 weeks | - Use with caution in patients with sinoatrial or atrioventricular heart block (may cause ventricular arrhythmias)  - Increases clearance of contraceptives. Women who use contraceptives are advised to use preparations containing at least 50 μg of ethinylestradiol, or switch to alternative contraceptive methods (Implanon, Depo-Provera, Mirena) in order to reduce the chance of pregnancy - High risk of teratogenicity. Cease therapy when pregnant.  - Use with caution in patients with hepatic impairment - A spectrum of hematologic effects have been reported with use ( agranulocytosis, neutropenia, leukopenia, thrombocytopenia, pancytopenia, and anemias) - Han-Chinese or Thai patients with the variant HLA-B*1502 may be at an increased risk of developing Stevens-Johnson syndrome and/or toxic epidermal necrolysis. |
| Gabapentin | 100-300 mg at bedtime or 100-300 mg 3 times daily | Increase by 100-300 mg 3 times daily every 1-7 d as tolerated | 3600 mg/d (in 3 divided doses) | 3-8 weeks (titration) plus 2 weeks at  maximum dose | - Reduce dose if impaired renal function - Consider lower starting dosages and slower titration in geriatric patients - Possible risk of teratogenicity. Consider whether benefits outweigh risks and start other drug if possible. - Little or average negative influence on driving performance. Comparable to blood alcohol content of 0,5–0,8‰. Advice: no driving in first week. |
| Pregabalin | 50 mg 3 times daily or 75 mg twice daily as tolerated | Increase to 300 mg/d after 3-7 d, then by 150 mg/d every 3-7 d as tolerated | 600 mg/d (200 mg 3 times or 300 mg twice daily) | 4 weeks | - Reduce dose if impaired renal function - Consider lower starting dosages and slower titration in geriatric patients - Possible risk of teratogenicity. Consider whether benefits outweigh risks and start other drug if possible. - Little or average negative influence on driving performance. Comparable to blood alcohol content of 0,5–0,8‰. Advice: no driving in first week. |
| Duloxetine (unlabeled use) | 30 mg once daily | Increase to 60 mg once daily after 1 week | 60 mg twice daily Note: Doses >60 mg/day administered in clinical trials offered no additional benefit and were less well tolerated than dose of 60 mg/day. | 8 weeks  Reevaluate therapeutic effect every 12 weeks | - Avoid use if CrCl <30 mL/minute or ESRD - Avoid use in patients with substantial ethanol intake, evidence of liver disease or hepatic impairment - May increase the risk of suicidal thinking and behavior (suicidality) in children, adolescents, and young adults with depression and other psychiatric disorders - Possible risk of teratogenicity. Consider whether benefits outweigh risks and start other drug if possible. - Little or average negative influence on driving performance. Comparable to blood alcohol content of 0,5–0,8‰. Advice: no driving in first week. |
| Venlafaxine (unlabeled use) | 37,5 mg once or twice daily | Increase by 75 mg each week | 225 mg/day | 4-6 weeks | - Mild to moderate hepatic impairment: reduce total daily dose by 50% - GFR: 10 to 70 mL/minute:  - Extended-release formulations: Reduce the total daily dose by 25% to 50%.  - Immediate-release formulation: Reduce the total daily dose by 25%.  - May cause significant increases in serum total cholesterol and triglycerides; monitor during long-term treatment - May cause sustained increase in blood pressure or tachycardia. Control pre-existing hypertension prior to initiation of venlafaxine.  - Use with caution in patients with recent history of MI, unstable heart disease, or hyperthyroidism. - May increase the risk of suicidal thinking and behavior (suicidality) in children, adolescents, and young adults with depression and other psychiatric disorders - Possible risk of teratogenicity. Consider whether benefits outweigh risks and start other drug if possible. |

*Abbreviations: CYP=cytochrome, CBC=complete blood count, HLA-B= human leukocyte antigen B, CrCl=Creatinine clearance, ESRD=end stage renal disease, GFR=glomerular filtration rate, MI=myocardial infarction*

*References:*

*- Dworkin. Recommendations for the Pharmacological Management of Neuropathic Pain: An Overview and Literature Update
- Grothe DR, Scheckner B, and Albano D, “Treatment of Pain Syndromes With Venlafaxine,” Pharmacotherapy, 2004, 24(5):621-29
- Locharernkul C, Loplumlert J, Limotai C, Korkij W, Desudchit T, Tongkobpetch S, Kangwanshiratada O, Hirankarn N, Suphapeetiporn K, Shotelersuk V. “Carbamazepine and phenytoin induced Stevens - Johnson syndrome is associated with the HLAB*1502 allele in Thai population.” Epilepsia, 2008 Dec;49(12):2087-91. doi: 10.1111/j.1528-1167.2008.01719.x. Epub 2008 Jul 14.x.
- www.farmacotherapeutischkompas.nl*

*- Byatt N, Deligiannidis KM, Freeman MP.* *Antidepressant use in pregnancy: a critical review focused on risks and controversies.* *Acta Psychiatr Scand. 2013 Feb;127(2):94-114. doi: 10.1111/acps.12042. Epub 2012 Dec 14.*

*- Denise S Hill, Bogdan J Wlodarczyk, Ana M Palacios, and Richard H Finnell. Teratogenic effects of antiepileptic drugs.* *Expert Rev Neurother. 2010 Jun; 10(6): 943–959.
- www.rijveiligmetmedicijnen.nl*
